# The Grants Recommended

**Published:** 1920-07-31

**Authors:** 


### THE GRANTS RECOMMENDED.

Acton Hospital, £2,500, to extension, new out-patient department, etc. Beckenham Cottage Hospital, £2,250, to extension, new operating theatre, etc. Charing Cross Hospital, £1,250, to improvements to *x*-ray department, nurses' home, etc. City of London Hospital for Diseases of the Chest, Victoria Park, £4,000, to surgical block and sanitary and other improvements. Croydon General Hospital, £1,000, to additional nurses' accommodation; subject to the provision of fire exit. Dreadnought Hospital, £7,000, to erection of sanatorium for tuberculous seamen, Bramshot, Hants. East Ham Hospital, £9,000, to rebuilding as general hospital. Finchley Cottage Hospital, £1,750, to extension, new administration block, etc. French Hospital, £2,000, to enlarged nurses' and servants' quarters, enlarged out-patient department, additional beds, and other improvements; in the order stated. Great Northern Central Hospital, £15,500, to new nurses' home, new boilers, and new kitchen, in the order stated. Guy's Hospital, £11,000, to nurses' home extension and special out-patient departments. Hampstead General Hospital, £1,000, to electrical and physical departments and other improvements. Hendon Cottage Hospital, £600, to new operating theatre, *x*-ray room, and improved nurses' accommodation. Hornsey Cottage Hospital, £1,500, to extension, new operating theatre, etc. Hospital for Consumption, Brompton, £7,000, to extension of sanatorium at Frimley. Hospital for Diseases of the Throat, £2,500, to rebuilding of amalgamated hospital. Hospital for Epilepsy and Paralysis, £2,000, to extension of out-patient department. King's College Hospital, £1,000, to equipment of wards, theatres, and special departments. London Homœopathic Hospital, £1,250, to improvements to boilers, lighting, lifts, heating, etc. London Hospital, £3,500, to new massage and electrical departments. London Jewish Hospital, £1,250, to extension of in-patient department, kitchen, operating theatre, etc. London Temperance Hospital, £6,000, to new nurses' accommodation, improvements to isolation ward, etc.; provided that the Hospital Committee are satisfied that the ward already vacant and the ward vacated by the scheme can both be opened to patients. Middlesex Hospital, £2,700, to new night nurses' home. Middlesex Hospital Cancer Charity, £300, to new night nurses' home. Mildmay Mission Hospital, £2,500, to fire-escape staircase, enlarged nurses' home, new operating theatre, and other improvements; on condition that the staircase is put in hand immediately. Miller General Hospital for South-East London, £15,000, to extension, new nurses' home, kitchen, and out-patient, casualty, and pathological departments. National Hospital for Diseases of the Heart, £2,000, to extension of out-patient department. National Hospital for the Paralysed and Epileptic, £1,000, to provision of out-patient operating-room, and improvements to in-patient theatre. Nelson Hospital for South Wimbledon, £2,500, to extension, additional nurses' rooms, etc. Norwood Cottage Hospital, £300, to additional rooms and new *x*-ray apparatus. Passmore Edwards Hospital for Willesden, £8,000, to extension as general hospital. Poplar Hospital, £6,000, to improved casualty department. Prince of Wales's General Hospital, £10,000, to extension, additional nurses' rooms, etc. Queen Mary's Hospital for the East End, £12,000, to new nurses' home and in-patient operating theatre; subject to the submission of block plan for ultimate utilisation of complete site. Royal Free Hospital, £8,500, to nurses' home and extension, in the order stated. Royal National Orthopædic Hospital, £24,000, to extension of out-patient department, *x*-ray, electrical, exercise, and Swedish-drill rooms, nurses' bedrooms, etc. St. Bartholomew's Hospital, £24,000, to new nurses' home. St. John's Hospital, Lewisham, £1,250, to new out-patient department. St. John's Hospital for Diseases of the Skin, £400, to new nurses' accommodation. St. Mark's Hospital, £600, to new nurses' rooms. St. Paul's Hospital for Skin, etc., £1,500, to new hospital in Endell Street. St. Peter's Hospital for Stone, £600, to new nurses' rooms, *x*-ray rooms, etc. St. Thomas's Hospital, £2,500, to enlargement of nurses' dining-room. University College Hospital, £500, to re-equipment of radiographic and electrical department. West End Hospital for Nervous Diseases, £2,500, to removal to Regent's Park, improved nurses' accommodation at Regent's Park, and reconstruction of out-patient department at Welbeck Street. Of the grant of £2,500, £500 is subject to the carrying out of the scheme for the provision of a new nurses' home at Regent's Park. Western Ophthalmic Hospital, £2,000, to reconstruction and extension. West London Hospital, £8,000, to new out-patient department. Wimbledon Hospital, £1,500, to new nurses' home, and resident medical officer's quarters. Woolwich War Memorial Hospital, £25,000, to building of general hospital. Total, £250,000.

